# Improving Therapy for Children with Scoliosis through Reducing Ionizing Radiation by Using Alternative Imaging Methods—A Study Protocol

**DOI:** 10.3390/jcm13195768

**Published:** 2024-09-27

**Authors:** Fee Keil, Robert Schneider, Nenad Polomac, Omar Zabar, Tobias Finger, Fabian Holzgreve, Marcus Czabanka, Christina Erbe, David A. Groneberg, Elke Hattingen, Daniela Ohlendorf, Panagiotis Diaremes

**Affiliations:** 1 Institute of Neuroradiology, Goethe University Hospital, 60528 Frankfurt, Germany; fee.keil@unimedizin-ffm.de (F.K.);; 2 Institute of Occupational, Social, Environmental Medicine, Goethe University, 60596 Frankfurt, Germanyholzgreve@med.uni-frankfurt.de (F.H.);; 3 Asklepios Katharina-Schroth-Klinik, 55566 Bad Sobernheim, Germany; 4 Department of Neurosurgery, Goethe University Hospital, 60596 Frankfurt, Germany; 5 Department of Orthodontics, Johannes Gutenberg University Hospital, Mainz, 55131 Mainz, Germany; erbe@uni-mainz.de; 6 Clinic for Orthopaedics, Goethe University Hospital, 60596 Frankfurt, Germany; panagiotis.diaremes@unimedizin-ffm.de

**Keywords:** pediatric radiation protection, scoliosis preoperative standard, follow-up, radiation exposure, video-raster stereography, interdisciplinary team

## Abstract

Background: Patients with scoliosis often require multiple imaging modalities. The aim of this study was to find out whether primary diagnosis, including surgical planning, could be carried out entirely without computed tomography (CT) scans and whether follow-up could be replaced with alternative methods without the use of X-rays. In order to reduce the radiation exposure in the diagnosis and treatment of severe scoliosis, we expect to replace X-rays with radiation-free or less-intensive radiation examinations. This study protocol is interdisciplinary. Methods: A total of 50 male and female patients (children and adolescents, aged 7–18 years) treated for scoliosis will be analyzed. In addition to routine projection radiographs, preoperative CT, and/or X-ray stereoradiography (EOS) examinations, thin-slice 3D magnetic resonance imaging (MRI) sequences will be retrospectively reformatted during the preoperative MRI examination. A three-dimensional back scan (video-raster stereography) and an intraoral scan will also be obtained. The following questions should be answered at the end of the project: (1) Can MRI examination with additional thin-slice 3D reconstruction answer all relevant questions for preoperative planning instead of CT? (2) Are EOS or whole-spine X-ray examinations in combination with MRI data sufficient for the evaluation of the pedicles and spinal deformity? (3) Does the Cobb angle in the radiograph correlate with the calculations from the back scanner image and can follow-up checks be replaced? (4) Are there any correlations between dental anomalies and scoliosis? Conclusions: Until now, pediatric patients with scoliosis have been diagnosed, monitored, and treated with numerous independent specialist disciplines, such as pediatricians, orthopedic surgeons, neurosurgeons, and general practitioners with different radiological issues. The aim of this project is to reduce radiation and lower perioperative risks by creating a preoperative and follow-up-related standard protocol in close interdisciplinary and targeted cooperation between all the specialist disciplines involved. In line with the holistic examination approach, the associated accompanying diseases and developmental disorders such as dental and neuronal malformations will also be examined. On the one hand, CT-based questions could be replaced with the reconstruction of thin-slice MRI sequences. In addition, it may be possible to use the three-dimensional back scan as an intermediate diagnostic procedure instead of X-rays in the monitoring of severe scoliosis. Insofar as correlations or causalities between scoliosis and occlusal anomalies, early orthodontic intervention could positively benefit the duration of therapy at a later stage.

## 1. Introduction

The clinical sign of scoliosis is a permanent pathological, three-dimensional axial deviation of the spine [1]. Zheng et al. [2] found a prevalence of idiopathic scoliosis of 2.4% in children aged 10–16 years, with girls being more frequently and severely affected [1,3]. X-ray projection and cross-sectional images are used to differentiate congenital scoliosis from structural idiopathic scoliosis and to classify AIS depending on the pattern and localization of the curvature [1,4]. Radiographic examinations with close follow-up are indispensable for the diagnosis and treatment decisions in patients with scoliosis [5]. For diagnosis, the degree of spinal curvature must exceed a Cobb angle of 10° in coronary view [1,6,7]. To determine the Cobb angle, it is necessary to obtain a whole-spine X-ray in the standing position. The Cobb angle is considered the gold standard and reliable for determining the extent of scoliosis and is assessed using a postero-anterior radiograph [1,8,9]. The risk of an increase in scoliosis increases the younger the patient and the higher the initial Cobb angle. In adolescents, a 10–20% probability of progression of scoliosis is found with a Cobb angle < 20° and of >70% with a Cobb angle > 20°. X-ray follow-up by means of projection images or EOS is performed at intervals of 6–12 months for Cobb angles greater than 30°. A conservative observational approach is common in patients with Cobb angles ≤ 20°; between 20 and 40°, nonsurgical therapy methods are used (i.e., physiotherapy, brace therapy); and, from ≥40 to 50°, surgical procedures are considered [1,8,10]. In addition to the initial diagnostic examinations and the preoperative imaging procedures, patients with scoliosis require numerous follow-up examinations to assess the course of the disease and, if necessary, to adjust the therapy concept, i.e., conservative vs. surgical.

According to curvature progression, King and the more recent Lenke classifications divide scoliosis into five and six main types, respectively, which have an influence on surgical planning prior to surgical treatment [11,12]. In addition to the preoperative exclusion of a Chiari malformation (type II), a tethered cord, and a syrinx, patients with planned surgery should also be excluded from the diagnosis of concomitant neurofibromatosis. A decisive point for the risk assessment of a worsening of the curvature is the skeletal maturity of the patient and the length growth that can still be expected. The degree of ossification of the iliac crest apophysis according to Risser [13] and the Sanders classification of the nondominant hand are used for radiological determination of bony maturity. The follow-up examinations are regularly performed with the aid of projection radiology examinations. In case of surgery for scoliosis, further diagnostic imaging procedures are required. For surgical planning, the following aspects have so far been clarified primarily by means of CT: determination of the pedicle thickness of vertebral bodies (for screw placement); detection of osseous stability- and growth-limiting anomalies, such as hemivertebrae and congenital vertebral defect. MRI is not a mandatory standard and is only used in specific cases to detect malformations of the brainstem, myelon, and nerves. Standardized MRI as part of primary diagnostics could detect neuronal malformations as well as incidental neuronal findings at an early stage, thereby reducing the risk of perioperative surgery.

An alternative noninvasive way of recording the geometry of the back is video-raster stereography. This method offers a reliable, radiation-free alternative to conventional radiography, especially in follow-up and screening examinations [14,15,16,17,18]. In this context, normal values of upper body posture (depending on age and sex) can be used as comparative values to identify pathological deviations [19]. The benefit of a two-dimensional photography [20] or video analysis [21] is the depth information that is calculated from the projected lines onto a curvature [16,22]. High intraclass correlation coefficients and good Cronbach’s alpha values for intra- and interday reliability for all spine parameters can be achieved when the markers are placed on defined anatomical landmarks [23,24,25,26]. This applies, among other things, to sagittal evaluation parameters, i.e., kyphosis or lordosis angle [18,27]. Additionally, a good intertester reliability of 0.979 was reported [18].

In addition, it appears that children with idiopathic scoliosis (AIS) have more frequent malocclusions than their healthy peers [28,29,30]. The interaction of neurophysiological or functional anatomical factors between dental abnormalities and posture has been demonstrated in previous studies [31,32]. Lippold et al., 2003 [33] found a statistically significant higher incidence of scoliosis (21.4%) in preschool children with Angle Class II dental malocclusion. Neck posture appears to be strongly related to the sagittal and vertical structure of the face [31]. Furthermore, there seems to be a significant relationship between the sagittal position of the mandible and a kyphotic posture [34].

To date, pediatric patients with scoliosis have been diagnosed, monitored, and treated with a range of independent specialist disciplines, including pediatricians, orthopedic surgeons, neurosurgeons, and general practitioners, each employing diverse radiological approaches. This project aims to reduce radiation exposure, minimize perioperative risks, and eliminate unnecessary duplicate examinations by establishing a standardized protocol for preoperative and follow-up care through close interdisciplinary and targeted collaboration among all involved specialists. Consistent with a holistic examination approach, this initiative will also encompass the assessment of associated comorbidities and developmental disorders, such as dental and neuronal malformations.

### 1.1. Rationale

As pediatric patients are involved, the main objective is to answer as many questions as possible preoperatively in order to avoid intraoperative surprises and to use alternative examination methods without additional (X-ray) radiation for follow-up examinations. In this context, we will investigate whether questions that could previously be answered through routine preoperative CT could also be answered by reconstructing thin-slice MRI sequences. In addition to the detection of spinal malformations by MRI in our study population, a comparison with the current literature will be conducted. We will also evaluate whether three-dimensional back scanning using light projections is suitable as an intermediate diagnostic procedure. For this purpose, the anatomical landmarks required for the back scan will remain marked during the X-ray and MRI measurements, so that the surface optometry readings obtained can be compared with the other evaluations. In addition, the influence of the examination position (supine in CT and MRI) as well as the deviation from the X-ray and back scanner standing images will be investigated. Furthermore, X-rays are taken preoperatively and, if necessary, according to the surgeon’s assessment, an EOS and a radiographic whole-spine image from the side and from the front are taken simultaneously. These are usually inadequately captured with routine cross-sectional images in patients with scoliosis. In addition to spinal changes, patients with scoliosis are more likely than patients without scoliosis to show concomitant temporomandibular pathology with jaw malalignments. To date, it is unclear to what extent these two pathologies are conditional; correlations have already been demonstrated but not causality. For the detection of temporomandibular malocclusions or the type of malocclusion, an intraoral scanner is used, with which an orthodontic model analysis can be carried out fundamentally in a very short time without any aids (such as plaster casts), without the need for further radiographs [28,29,35].

### 1.2. Study Questions

(1) Can MRI examination with additional thin-slice 3D reconstruction answer all relevant questions for preoperative planning instead of CT? Are EOS or whole spine X-ray examinations in combination with MRI data sufficient for the evaluation of the pedicles and spinal deformity?
(2) Does the Cobb angle in the radiograph correlate with the calculations from the back scanner image and can follow-up checks be replaced by this video-raster stereography measurement (radiation-free)?
(3) Are there any correlations between dental anomalies (e.g., crossbite) and scoliosis?

## 2. Materials and Methods

### 2.1. Study Design and Setting

This will be a prospective, nonrandomized trial with patients with scoliosis. All subjects with severe scoliosis will be recruited from the outpatient clinic of the orthopedic department of the University Hospital Frankfurt. In addition to routine projection radiographs and preoperative CT scans that are available in some cases and/or X-ray EOS scans, special 3D thin-slice MRI sequences will be retrospectively reformatted during the required preoperative MRI scan for all study participants, as well as a three-dimensional back scan and an intraoral scan. All procedures and scoliosis parameters obtained will be compared and correlated.

### 2.2. Participants/Study Subjects/Sample Size Calculation

The sample size calculation will be based on our primary hypothesis. To evaluate whether thin-slice MRI examination with reconstruction can adequately address the relevant questions for preoperative planning instead of CT, we will determine the Wanatabe classification for pedicle type based on both MRI and CT data and calculate Cohen’s kappa. Our goal is to demonstrate a Cohen’s kappa of more than 0.4 (indicating at least moderate agreement according to standard classification) using a one-sided test with a significance level of alpha = 5%. For sample size planning, we will simplify our assumptions by considering that the categories are approximately equally populated and that there is, in fact, at least substantial agreement with a kappa of 0.75. In this scenario, at least 30 patients will be sufficient to achieve a statistical power of 95%, even with a 5% drop-out rate. A relatively high power was chosen to account for an uneven distribution and to ensure sufficient statistical power overall. For the correlation tests involving quantitative measures of the secondary objectives, we also expect adequate statistical power. In general, statistical tests will be conducted with a two-sided approach and a significance level of alpha = 5%, except for the primary objective.

Within the framework of the project, at least 50 male and female children and adolescents aged > 7 to 18 years will be analyzed (according to the calculated power analysis). All of them will present to the Orthopaedic Clinic of the University Hospital Frankfurt with a clinical picture of scoliosis between 2022 and 2025. Height and weight will be recorded at admission. In addition, a separate medical history form will be used to record any previous conservative therapies (orthotics, braces, physiotherapy), including the type and duration of treatment. The following criteria apply for inclusion in this study:

- Children/adolescents diagnosed with severe scoliosis by major curve in the X-ray > 40° [36];
- Planned MRI examination of the spine at the Institute of Neuroradiology;
- An EOS and/or a CT examination of the spine has already been performed as part of the diagnosis or must still be planned independent of the present project;
- Children must be able to cooperate (>7 years, no motor or cognitive impairment);
- Consent of the legal guardians as well as of the child.
- The following criteria apply for exclusion from this study:
- There will be no upper curve limit as an exclusion criterion;
- Previous spinal surgery;
- Mental and/or physical impairments that do not warrant a 30 min MRI scan or contraindications to an MRI scan (i.e., implants, pregnancy).

Informed consent from a parent and/or legal guardian for study participation will be obtained.

The collected data will be stored in a pseudonymized format, in accordance with the local data security policy and in full compliance with GDPR. A specific data protection plan, overseen by the clinic’s data protection officer, will be implemented. The data will be stored in secure, segregated folders, with access restricted to authorized personnel only (e.g., the principal investigator, study physicians, and examiners), who will have the ability to link the data to individual patients through the pseudonymization list. Data from the study will be made available in future publications in anonymized form, in compliance with ethical guidelines, and upon request, according to journal policies and legal requirements. Only the principal investigator, study physicians, and examiners will be able to link the collected data to specific names/patients using a written list. A study folder will be created for each patient, containing the score sheets, which will be securely stored in the senior physician’s office. The transfer of any personal data to other individuals is not planned. All data will be retained for 30 years.

This study was approved by the medical ethics board for research involving human subjects of the Goethe University (2022-611) in Frankfurt am Main, Germany.

X-ray: X-ray images are used to determine the extent of the scoliosis by measuring the Cobb angles Philipps GmbH (Hamburg, Germany). Two straight lines are drawn at the base and top plates that are tilted the most relative to each other. The angle created at the point of intersection corresponds to the Cobb angle. The two vertebrae are called end vertebrae. At the center of the curvature, the vertebra with the maximum rotation is defined as the apical vertebra or apex. If there is also an opposing curvature corresponding to a smaller secondary curvature, this Cobb angle is determined analogously. In addition, the iliac crests should be visualized in the radiograph [1]. This is used to assess the ilium apophysis and thus to determine Risser’s sign. The lateral to medial ossification of the apophysis is determined and divided into different stages [37]. Yang et al. [38] demonstrated strong intraobserver variability. To correctly classify Risser’s sign and prevent errors, they developed a modified staging system [38]. A more reliable method of assessing the remaining growth potential of the spine is the Sanders hand classification. The intra- and interobserver reliability of Sanders and its predictive value in the progression of scoliosis make it a very valuable tool in assessing spinal growth [39].

The most important classification in surgical planning of scoliosis performed on the basis of radiographs (sagittal and coronal) is the Lenke classification [1,12]. The Lenke classification is a system used to classify and describe the types of scoliosis curves seen in adolescent idiopathic scoliosis (AIS). The Lenke classification system divides AIS into three categories based on the location of the curve in the spine: thoracic curves (T) located from the 1st to the 11th thoracic vertebrae; thoracolumbar curves (TL), located in the middle of the spine, from the 12th thoracic vertebra to the 1st lumbar vertebra; lumbar curves (L), located from the 2nd to the 5th lumbar vertebrae. The Lenke classification system is used by spine surgeons and orthopedic specialists to help guide treatment decisions for AIS. Treatment options may include bracing, spinal fusion surgery, or observation depending on the severity of the curve and the age of the patient. The Lenke classification system helps physicians to determine the appropriate treatment plan based on the specific characteristics of the curve. In addition, functional radiographs can be taken according to bending [40]. These are taken in maximum lateral bending to the right and to the left in order to estimate the flexibility of the spine and the potential maximum possible correction [41,42]. Also, on the basis of these radiographs, the primary curvature can be differentiated from the secondary curvature in the case of several approximately equal Cobb angles. 

EOS: The EOS system EOS Imaging (Paris, France) is a medical imaging technology developed by EOS imaging. The system uses a proprietary technology called slot-scanning, which involves capturing two simultaneous X-ray images from different directions as a patient stands or sits in an upright position. This technique provides high-quality, low-dose 2D and 3D images of the skeletal system, particularly the spine and lower limbs, with less radiation exposure compared to that of traditional CT scans. One of the main advantages of the EOS system is its ability to provide full-body images of patients in an upright position, which can be particularly useful for assessing conditions such as scoliosis or assessing the alignment of lower limb joints. The system also provides high-resolution images, which can aid in the diagnosis and treatment planning of orthopedic conditions [43].

MRI: The MRI device used will be a 1.5 Tesla device (Ingenia) from Philipps GmbH (Hamburg, Germany). The sequences to be used consist of an axial and a sagittal T2w, a coronary stir, a sagittal T1w, and an axial T1w with and without fat saturation, if applicable. Furthermore, an isovoxel 3D T2 sequence, with 1 mm slices, will be reformatted in all spatial directions following the examination. 

**Figure 1:**
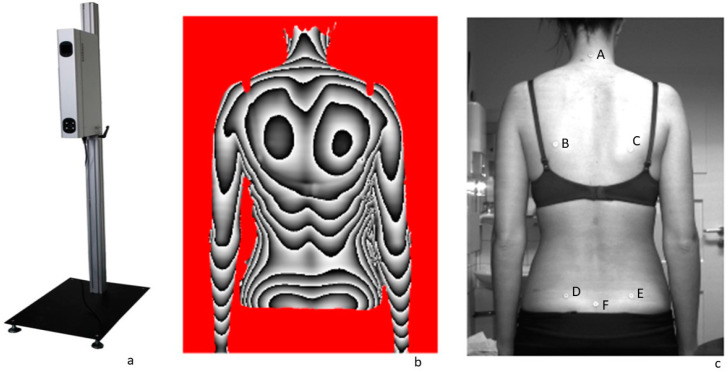
(**a**) MiniRot Combi back scanner (ABW GmbH, Frickenhausen/Germany), (**b**) three-dimensional phase picture of the back, (**c**) illustration of the exact marker position on the back: A: vertebra prominens (7th cervical vertebra), B: left lower scapular angle, C: right lower scapular angle, D: left spina iliaca posterior superior (SIPS), E: right spina iliaca posterior superior (SIPS), F: sacrum point (cranial beginning of the gluteal cleft).

Radiation-free back scanner: The three-dimensional back scanner “BodyMapper” (ABW GmbH, Frickenhausen, Germany) records changes in the upper back posture while standing by using video-raster stereography. The sampling frequency is 50 Hz with a spatial resolution of 1/100 mm. Marking of six anatomical fixed points on the bare back is required to generate the evaluation parameters. These anatomical landmarks are shown in Figure 1. In comparison with other methods, Asamoah et al. [44] found it to be effective in diagnosing scoliosis and certain deformations. Values for sensitivity and specificity were 98% and 84%. Other authors [45] reported good correlations with angle measurements using video-raster stereography and X-ray (r > 0.8 to 0.93). Regarding evaluation criteria of the upper body posture, all calculated evaluation parameters can be divided into three parts: the spine (marker on C7 and Rima Ani), shoulder (marker at the highest place of the left/right scapula), and pelvis area (marker on left and right spina iliaca posterior superior [SIPS]). Details were already described by Ohlendorf et al. [46].

Intraoral scan: An intraoral scan will be performed using an intraoral scanner (Primescan, Sirona Dentsply, Bensheim, Germany), and the resulting 3 -model will be analyzed using software (OnyxCheph^3TM^ Version 3.2.223 (608), Image Instruments GmbH, Chemnitz, Germany). In all three dimensions (anteroposterior, transverse, and vertical), occlusal conditions, malocclusion, and dental anomalies will be examined, i.e., transverse: crossbite or scissor bite; anteroposterior: overjet and angle classification; vertical: overbite, open bite or deep bite.

### 2.3. Measurement Protocol

**Figure 2:**
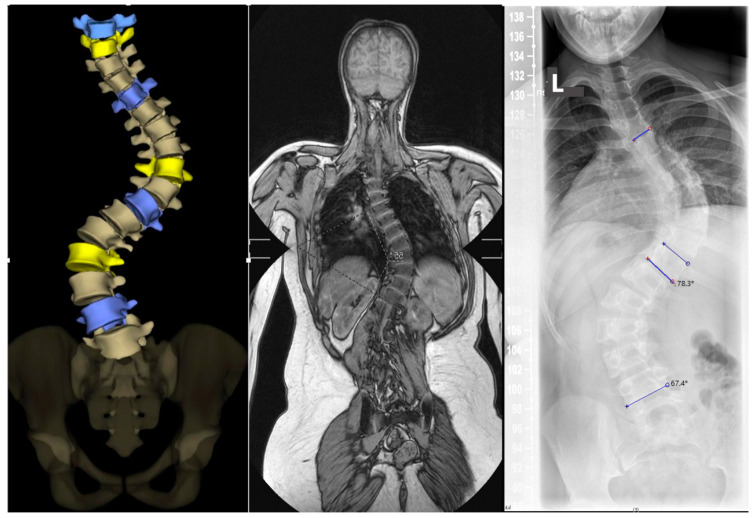
From left to right: EOS, MRI scout image, whole-spine X-ray. (**Left image**) Coronary EOS view, with color-coded apical vertebrae in yellow and neutral vertebrae in purple. (**Middle image**) Coronary MRI image in T1W sequence for planning the thin-layer 3D sequences. (**Right image**) X-ray standing image for determining the Cobb angle. The blue lines are reference lines for calculating the angles. The upper line is for the thoracic curve, the two middle lines for the major curve and the lower line for the lumbar curve.

Routine diagnostics: Following the initial scoliosis diagnosis, which will include X-rays in two planes, additional routine standardized examinations with functional images and, if necessary, 3D imaging using EOS X-rays of the spine and/or if deemed necessary a preoperative CT of the spinal axis will also follow within this study (Figure 2). The indication for MRI examination to exclude congenital malformations will be a precondition for this study (Figure 2).

Additional protocol: After inclusion in this study, anatomical landmarks will be marked on the patient’s back for all subsequent examinations and visualized using different markers in the corresponding examination modality. EOS image: Additionally, visible markers will be glued to each of the six markers of the back scan using adhesive tape so that the same reference markers will also be available here for evaluation purposes. MRI-image: Liquid-containing capsules will be taped onto the markers of the back scan instead of metal markers during the X-ray. In cases of splitting the measurement over two days, the X-ray image will be taken first. For this purpose, the markers of the back scan will then be marked with a permanent marker so that the same position can be used again the next day for the rest of the measurements. Additional diagnostics: The two additional examinations by means of the back scanner and the oral scanner will take a total of approx. 15 min and will be coordinated with the MRI examination in terms of time and space. All preoperative examinations will be performed at close intervals (within 7 days) to minimize the risk of measurement deviations due to disease progression. Back scan: For this purpose, six anatomical landmarks will be marked on the unclothed back [46]. All patients will stand in habitual body posture at a predefined distance of 90 cm with the back to the scanner. The arms should hang loosely next to the body with the gaze directed straight ahead in order to exclude changes in head posture. One measurement will last 5 s and will be repeated 5 times with short periods of rest in between. Intraoral scan: Immediately before the MRI examination is performed, the dentition will be scanned in addition to the back scan. For this purpose, the subject will sit on a chair with the body upright and the head straight. Postoperative routine diagnostics: In the case of surgery, an X-ray and/or EOS will be performed as standard procedure following surgery at the discretion of the surgeon depending on the clinical course, and, in the case of postoperative complaints, a CT could be performed to demonstrate the screw position. In addition, the anatomical landmarks should be marked with visible markers, and the back scan should be performed again on the day of the postoperative X-ray or EOS examination, taking an additional 5 min for the patient.

Statistical data analysis

Descriptive statistics and exploratory comparisons between measurement methods will be calculated from the following variables:

- Demographics: Age, height, weight, sex, Body Mass index (BMI).
- Spinopelvic parameters: Lumbar lordosis, pelvic incidence, pelvic tilt, sacral slope, pelvic incidence–lumbar lordosis mismatch.
- Patient clinical parameters (pelvic/shoulder low, rib hump, lumbar bulge).
- Classification of pedicles to be instrumented according to Wanatabe.
- Cobb angle (thoracic, lumbar).
- EOS parameters (pelvic obliquity, rotation of the apical vertebra).
- Kyphosis and lordosis angle.
- Risser and Sanders stages (growth still to be expected).
- Appearance of scoliosis according to Lenke and King.

In particular, nonparametric or even parametric correlations will be determined, including Bland–Altman regression analyses for quantitative variables and Cohen’s kappa for nominal variables, for comparison between parameters by alternative examination methods without X-rays compared with CT-based parameters (Table 1).

To verify whether thin-slice MRI examination with reconstruction can answer our relevant questions for preoperative planning instead of CT, King or Watanabe classifications of curvature progression will be determined from both MRI data and CT, and Cohen’s kappa will be calculated for this purpose. The aim is to demonstrate a Cohen’s kappa of more than 0.4 (at least moderate agreement according to the standard classification) with a one-sided test and significance level of alpha = 5%. In principle, the statistical tests should be two-sided with a significance level of alpha = 5%, with the exception of that for the main test objective.

## 3. Results

1. Thin-slice 3D MRI examination with reconstruction can answer all relevant questions for preoperative planning instead of CT. EOS or X-ray examination in combination with MRI data is sufficient for imaging pedicles and spinal deformity.

We expect to be able to replace preoperative CT diagnostics and obtain further information by incorporating an additional 3D MRI sequence. We anticipate that through this method, using MRI in combination with X-ray or EOS data, we will be able to detect and assess both preoperative and diagnostic parameters in a single examination. This includes parameters such as pedicle thickness according to Wanatabe, degree of disc degeneration, intraspinal anomalies, as well as formation and segmentation disorders of the vertebral bodies. Our goal is to completely eliminate the need for preoperative CT examinations by relying solely on MRI.

2. The Cobb angle in the radiograph correlates with the calculations from the back scanner image, and follow-up checks can be replaced by a three-dimensional back measurement (radiation-free).

The parameters obtained from the back scanner regarding the sagittal profile of the spine have already been validated in the general population [19]. In the context of our study, we expect a correlation between the data from the back scanner and the data from EOS and X-ray imaging, enabling the evaluation of the coronal plane and the Cobb angle in patients with severe scoliosis.

3. Correlations between dental anomalies (e.g., crossbite) and scoliosis can be proven [31,32,33,34].

We expect to find existing correlations between upper body posture and bite position, particularly in patients with severe scoliosis.

## 4. Discussion

So far, there is no standard for the preoperative diagnosis of juvenile patients with scoliosis. If scoliosis is suspected, X-ray images of the spine are obtained [40,47,48,49,50]. These allow the scoliosis form to be classified and the Cobb angle to be determined [51]. Depending on the extent of the scoliosis, a surgical indication is made. Currently, it is up to the surgeon to decide what type of imaging is needed preoperatively. It is the surgeon’s individual decision whether functional images, computed tomography, or magnetic resonance imaging are required for surgical planning and to exclude underlying or concomitant malformations or space-occupying lesion [40,49,52]. The assessment of the bone with the question of screw-bearing pedicle thickness, hemivertebra to be resected, or partial vertebral body fusion has so far been performed by computed tomography but could possibly also be clarified by MRI with appropriate follow-up. Less severely affected patients with milder degrees of scoliosis may also benefit from alternative imaging modalities and preoperative MRI as intraoperative risks can be reduced. Although they rarely require a CT scan initially and can often be treated with conservative therapies, their response to therapy needs to be closely monitored with follow-up examinations, which have previously included radiographic imaging. According to the Federal Office for Radiation Protection, this would save approximately 770 µGym^2^ per X-ray examination, which can be dispensed with by using a back scan, among other things.

If surgical therapy is planned, concomitant anomalies should be excluded before spinal stabilization. In particular, dysraphic anomalies (e.g., cleft medullary malformations, spinal lipoma, fatty filum terminale, secondary tethered cord syndrome), which are highly likely to cause clinically relevant tethered cord syndrome, can be detected with preoperative MRI examination, which has not been performed as the standard so far, and subsequently taken into account for surgical planning. Developmental anomalies in the craniocervical junction (e.g., Chiari type I and II malformations, basilaris intussusception) can cause severe neurological deficits, especially in the absence of preoperative knowledge during surgical scoliosis treatment, if not taken into account during intubation or head positioning, and can be easily detected with our standard MRI examination. Patients with nerve sheath tumors or other causes of significant spinal stenosis in a pediatric patient cohort must also be diagnosed prior to any spinal intervention. Adequate MRI imaging is a prerequisite for significantly reduced intraoperative risk in young patients already characterized by a spinal deformity. Close interdisciplinary cooperation between neuroradiology, orthopedics, and pediatric neurosurgery is key to the success of this project and the smooth and safe treatment of our young patients with a mid- to long-term aim of establishing this kind of set up as the gold standard for treatment and diagnostics for juvenile patients with scoliosis. The initial step is always to take a detailed medical history, including family history, and, in female patients, to determine the time of menarche, if possible. With the onset of menarche, residual skeletal growth can be expected for a further two to two and a half years. In boys, an equivalent process takes place about two years after the onset of voice change [53,54]. The clinical examination follows the anamnesis. This includes, among others, a survey of the mobility of the spine in the form of the forward bending test according to Adams with the use of a scoliometer [49,55]. The scoliometer has a high interobserver reliability [40,47,48,49,50]. Whereas Coelho et al. [50] found a meaningful association with the radiologically determined Cobb angle, other authors described an investigator or BMI dependence [55]. In summary, the aim of the present project is to replace the above-mentioned scoliosis analysis parameters with alternative examination procedures without X-rays. Taking into account the increased sensitivity of children to radiation and its potential long-term consequences, alternative procedures without the use of ionizing radiation are particularly desirable [56]. In particular, CT examinations in childhood have been shown to increase the risk of leukemia and triple the risk of developing a brain tumor. Particularly in cases of disease requiring intermediate or follow-up examinations, the procedure of video-raster stereography and the associated visualization of spinal topography offers a potential, noninvasive alternative, because video-raster stereography has been shown to be a valuable tool to determine back geometry [19]. If the back scanner is suitable as an intermediate diagnostic procedure in the future, it could also support medical diagnostics in countries where financial resources play an even greater role. According to Ohlendorf et al. [46], studies have been published regarding the normal values of upper body posture as a function of age decade (starting at 20 years) and sex using this same measurement system [19]. In summary, men and women have a balanced, upright, and symmetrical upper body posture regardless of age. Those standard values can help in decision making for therapeutic or clinic interventions as well as in the evaluation of therapeutic interventions. Therefore, they could also be used as reference values for the present project, and, if necessary, back scanners could be used for screening examinations in adolescents. Of course, bite location data are not used in providing less radiation or safer scoliosis surgery. However, analyses of the correlation between temporomandibular malpositions and upper body posture would also help (dental) practitioners diagnose anomalies of the temporomandibular system (e.g., crossbite, temporomandibular joint disorders) and in the dorsal upper body posture (e.g., scoliotic malposition, kyphotic developments in the thoracic spine) at an early stage in order to prevent progression.

### Limitations

For both the three-dimensional back scanner and the introaral scanner, studies have mainly focused on adults [19]. Age-related values of the upper body posture for children and adolescents are not currently available and will be collected in another project. Results are expected before this study is completed. One problem is the age of the subjects during the MRI. The younger the patient, the lower the fat content in the bone and, consequently, the more reduced the signaling. Consequently, the differentiation of ligaments, membranes, and bones could be more difficult. Also, the duration of the examination could be problematic, especially in younger patients, as they have to lie still for about 30 min in the MRI. This can lead to motion artifacts and measurements having to be repeated or not being able to be evaluated.

We acknowledge that this study protocol primarily focuses on detailing the methodology of our project. While initial results would undoubtedly add valuable context, the complexity and multifaceted nature of our study necessitate a dedicated and thorough description of the methods. Combining this with the results in a single manuscript could obscure the holistic and interdisciplinary approach that is central to our research. To address this, we present this study protocol to offer a comprehensive overview of our prospective clinical research project. By doing so, we aim to enhance transparency in prospective clinical research and reduce publication bias by ensuring the reproducibility of our study design and analysis.

## 5. Conclusions

In this interdisciplinary study, the above-mentioned routine scoliosis parameters will be compared with measured values from alternative examination procedures without the use of ionizing radiation in the diagnosis as well as in the conservative and operative treatment of severe scoliosis. In addition, the increasingly important holistic approach will be considered through correlation calculations between anomalies in the temporomandibular system and the spine.

## Figures and Tables

**Table 1:** Relevant variables.

Demographic Variable	Confirmation of Target Values through New Procedures	Nominal or Quantitative	Range
Age, height, weight, sex, body mass index (BMI)	MRT vs. CT: classification of pedicles to be instrumented according to Wanatabe.	Nominal	A, B, C, D
	X-ray vs. back scan: Lumbar lordosis, pelvic incidence, pelvic tilt, sacral slope, pelvic incidence–lumbar lordosis mismatch	Quantitative	20 ± 40°
	Cobb angle (thoracic, lumbar)	Quantitative	20–100°
	EOS vs. back scan: pelvic obliquity, rotation of the apical vertebra Appearance of scoliosis according to Lenke and King	Quantitative Quantitative Nominal	0 ± 3 cm 0 ± 30° 1–6/A, B, C/+, N, -
	X-ray vs. MRT: Risser/Sanders stages (growth still to be expected)	Nominal	1–6/1–8
	Radiation dosis	Quantitative	mGy/m^2^

## Data Availability

Not applicable.
